# Colony‐stimulating factor 1 positive (CSF1^+^) secretory epithelial cells induce excessive trophoblast invasion in tubal pregnancy rupture

**DOI:** 10.1111/cpr.13408

**Published:** 2023-01-31

**Authors:** Xiaoya Zhao, Li Yan, Sifan Ji, Yiqin Zhang, Lisai Ha, Chuqing He, Yuan Tian, Luting Chen, Qian Zhu, Mingqing Li, Jian Zhang

**Affiliations:** ^1^ Department of Obstetrics and Gynecology, International Peace Maternity and Child Health Hospital, School of Medicine Shanghai Jiaotong University Shanghai China; ^2^ Shanghai Municipal Key Clinical Specialty Shanghai China; ^3^ Department of Assisted Reproduction, International Peace Maternity and Child Health Hospital, School of Medicine Shanghai Jiaotong University Shanghai China; ^4^ Hospital and Institute of Obstetrics and Gynecology, Shanghai Medical College Fudan University Shanghai China

## Abstract

Tubal ectopic pregnancy (TEP) occurs when an embryo aberrantly implants in the fallopian tube, leading to abortive or ruptured tubal ectopic pregnancy (AEP or REP). Poor outcomes of REP include maternal infertility or mortality. Current studies on the prevention and treatment of ruptured tubal ectopic pregnancy (REP) are unfortunately hampered by a lack of the cell spectrum and cell–cell communications in the maternal–foetal interface. Here, we investigate the mechanisms of tubal rupture through single‐cell transcriptome profiling of the fallopian tube‐trophoblast interface in REP, AEP and intrauterine pregnancy patients. In REP, extravillous trophoblast (EVTs) cells form a dominant cell population, displaying aggressive invasion and proliferation, with robust differentiation into three subsets. Cell communication analysis identified colony‐stimulating factor 1 (CSF1), overexpressed by fallopian tube secretory epithelial cells in REP, with CSF1R on EVTs and macrophages, as a ligand/receptor pair that stimulates EVT invasion and macrophage accumulation. CSF1+ secretory epithelial cells stimulate EVTs migration and invasion, leading to a tubal rupture in REP. These results provide a mechanistic context and cellular milieu leading to tubal rupture, facilitating further study and development of therapeutics for REP in early pregnancy.

## Graphical abstract

Diagram of the main receptors and ligands expressed on the secretory epithelial cells, extravillous trophoblasts (EVTs) and macrophages that are involved in invasion and cellular recruitment. REP, ruptured tubal ectopic pregnancy. AEP, abortive tubal ectopic pregnancy.

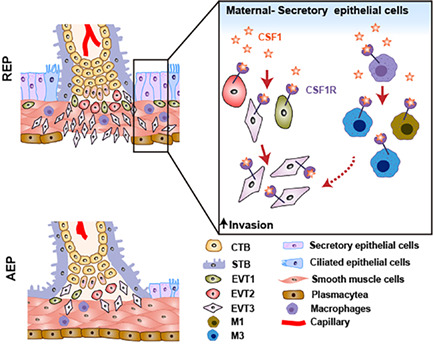

## 1 INTRODUCTION

Ectopic pregnancies (EPs) result from embryo implantation at sites other than the uterine endometria (intrauterine pregnancy, IP), 98% of which occur within fallopian tubes.^1^ This can lead to infertility and/or even death in affected patients, and accounting for 75% of early pregnancy‐related deaths.^2^, ^3^, ^4^ In clinical practice, the actual morbidity rate may be even higher.^5^, ^6^ Upon implantation in the fallopian tube, tubal EPs (TEPs) can evolve into either abortive or ruptured EPs (hereafter termed AEP and REP, respectively). Both are diagnosed and confirmed by a combination of beta‐subunit human chorionic gonadotropin (β‐hCG) dynamics, ultrasonography and laparoscopy.^7^ AEPs are typically characterized by low and gradual declining β‐hCG dynamics and haemodynamic stability with intact fallopian tubes and are therefore monitored at home or treated with methotrexate as necessary, a widely used and cost‐effective intervention that preserves patient fertility.^7^ By contrast, REP, the most common cause of maternal mortality among all tubal EPs, is commonly characterized by elevated β‐hCG levels, an active foetal heart, and haemodynamic instability.^8^, ^9^ REPs thus require more invasive management, such as emergency laparoscopic resection of the fallopian tube and foetus. While the factors associated with increased risk of tubal rupture have been intensely investigated, such as maternal age (≥35), implantation in the isthmus and >5000 IU/L of β‐hCG,^9^, ^10^ it remains uncertain whether these risk factors in REP are causal, due a lack of mechanistic understanding of the distinct events leading to AEP and REP.^11^, ^12^, ^13^

The human placenta is a highly specialized and multifunctional organ that is essential for foetal growth and survival. Three placenta‐specific cell types are responsible for its primary functions, including villous cytotrophoblasts (vCTBs, the stem cell population in trophoblasts), syncytiotrophoblasts (STBs), and extravillous trophoblasts (EVTs).^14^ VCTBs further differentiate into two lineages that either fuse to an external layer of multinucleated STB or undergo an epithelial‐mesenchymal transition to give rise to EVT cells.^14^ After implantation, EVTs display a migratory and invasive phenotype, ensuring the success of the pregnancy by anchoring the placenta to the uterus.

The aberrant regulation of EVT invasion can lead to a series of obstetric syndromes. Incomplete invasion is associated with pre‐eclampsia, foetal growth restriction (FGR) and stillbirth,^15^, ^16^ while excessive invasion results in placental accreta and choriocarcinoma.^17^ Histopathology studies have demonstrated that tubal rupture is positively correlated with the depth of EVT invasion.^18^, ^19^ Invasion of the fallopian tube wall by EVT can potentially compromise its structure and disrupt its functions in pregnancy by inducing an inflammatory response.^18^ In IP, several factors secreted from decidua, uterine smooth muscle cells and decidual natural killer (dNK) cells act as the key regulators of EVT function.^17^, ^20^ In addition, macrophages are the predominant immune cells at the maternal–foetal interface in EP, and the classically activated phenotype (Macro1) promotes trophoblast apoptosis and inhibits trophoblast invasion, resulting in tubal abortion.^21^, ^22^ Thus, an imbalance in trophoblast invasion provides obvious evidence of the pivotal regulatory contribution of the maternal–foetal interface microenvironment.^23^

Under certain physiological conditions, the motion of tubal cilia, smooth muscle contraction and tubal secretory fluids play a central role in tubal transport. Altered cilia function, a perturbed chemotactic tubal microenvironment and dysregulation of migration by the fertilized egg can all result in stranding the embryo in the tube, and consequently, tubal pregnancy.^24^, ^25^ However, after implantation, the cellular interactions within the fallopian tube that regulate EVT functions and the mechanisms leading to different pregnancy outcomes are still unclear.

Here, we conducted single‐cell sequencing to comprehensively determine the cell states and subtypes involved in the development of tubal rupture and maternal–foetal communication in the narrow space of fallopian tubal walls. Bioinformatic analyses were used to evaluate the functions of different cell types and subtypes, and generate detailed molecular and cellular maps of the human fallopian tubal–placental interface. These analyses provide an in‐depth understanding of tubal rupture at the single‐cell level and can help guide the development of novel therapeutic strategies to combat the aetiology of REP.

## 2 METHODS

### 2.1 Patient samples

All the maternal–foetal interface samples from normal intrauterine pregnancies (IP, *n* = 14), abortive tubal EPs (AEP, *n* = 54) or ruptured tubal EPs (REP, *n* = 59) at gestational weeks 6–8 were collected immediately after surgically resected chorionic villi and fallopian tube/uterine decidua. Then, transported tissues from the operation room to laboratory within 15 min. Voluntarily terminated intrauterine pregnancies that confirmed by ultrasonography combined with blood test or urine pregnancy test. For EP placental villi (including AEP and REP), the tubal implantation was first diagnosed by ultrasonography, followed by confirmation and removal by salpingectomy. All EP samples located in the ampulla of fallopian tube and were collected without receiving methotrexate treatment. AEPs are usually diagnosed by low β‐hCG levels and haemodynamic stability of unruptured fallopian tubes.^26^ While REPs usually were defined high β‐hCG level, active foetal hearts and maternal haemodynamic instability.^9^ Exclusion criteria for all samples are IP with EP histories, miscarriage, preeclampsia, preterm delivery. Some risk factors associated with EP, for example, smoking, obvious tubal inflammatory adhesions, previous fallopian tubal diseases and tubal surgery histories, were also excluded for avoiding complications. In each case, gestational days (GDs) were confirmed from the last menstrual period. Details about distribution of samples according to baseline characteristics are provided in Table 1.

**TABLE 1:** Details of baseline characteristics of all clinical samples. [Correction added on 22 February 2023, after first online publication: Table 1 has been corrected].

Samples	Age (years)	Gestational age (days)	Gravidity	Parity	HCG (IU/L)	Viable fetus	Method
AEP01	38	43	2	1	609	NO	scRNA‐seq
AEP02	35	51	2	1	788	NO	scRNA‐seq
AEP03	26	54	1	0	360	NO	IHC
AEP04	34	59	2	1	1636	NO	IHC
AEP05	25	54	0	0	1501	NO	IHC
AEP06	28	55	0	0	854	NO	IHC
AEP07	36	44	1	1	1285	NO	IHC
AEP08	36	36	2	1	447	NO	IHC
AEP09	35	57	3	1	830	NO	IHC
AEP10	34	53	1	1	1426	NO	IHC
AEP11	33	53	2	1	338	NO	IHC
AEP12	29	55	0	0	1425	NO	IHC
AEP13	31	36	0	0	351	NO	IHC
AEP14	27	46	1	0	466	NO	IHC
AEP15	26	42	0	0	603	NO	IHC
AEP16	32	43	1	0	1928	NO	IHC
AEP17	30	43	2	0	1824	NO	IHC
AEP18	37	40	1	1	1897	NO	IHC
AEP19	37	47	1	1	388	NO	IHC
AEP20	31	43	1	1	674	NO	IHC
AEP21	30	45	1	0	864	NO	IHC
AEP22	29	42	1	1	503	NO	IHC
AEP23	23	51	0	0	1031	NO	IHC
AEP24	39	56	3	1	227	NO	IHC
AEP25	32	46	1	1	1979	NO	Explant
AEP26	36	59	1	1	1040	NO	Explant
AEP27	30	52	1	1	846	NO	Explant
AEP28	34	49	0	0	1712	NO	Explant
AEP29	43	52	2	1	1596	NO	Explant
AEP30	28	42	1	1	1995	NO	Explant
AEP31	29	49	0	0	1958	NO	Explant
AEP32	28	53	1	1	1940	NO	Explant
AEP33	37	48	2	2	1799	NO	Explant
AEP34	39	56	2	1	1021	NO	Explant
AEP35	32	46	1	0	848	NO	Explant
AEP36	28	48	0	0	1736	NO	Explant
AEP37	37	43	3	1	1672	NO	Explant
AEP38	29	47	1	1	602	NO	IF
AEP39	26	45	1	0	789	NO	IF
AEP40	31	43	2	0	1119	NO	IF
AEP41	34	46	1	1	792	NO	IF
AEP42	25	49	0	0	645	NO	IF
AEP43	33	50	1	0	1201	NO	IF
AEP44	33	51	1	0	589	NO	IF
AEP45	31	55	1	0	1207	NO	IF
AEP46	37	42	1	1	1845	NO	IF
AEP47	32	46	1	0	1847	NO	IF
AEP48	29	53	2	1	1503	NO	IF
AEP49	32	42	1	0	178	NO	Flow cytometry
AEP50	36	59	2	1	425	NO	Flow cytometry
AEP51	36	55	1	1	1489	NO	Flow cytometry
AEP52	34	45	3	1	1037	NO	Flow cytometry
AEP53	24	42	1	0	1022	NO	Flow cytometry
AEP54	38	44	1	0	1303	NO	Flow cytometry

Samples	Age (years)	Gestational age (days)	Gravidity	Parity	HCG (IU/L)	Viable fetus	Method
REP01	31	41	1	0	3848	YES	scRNA‐seq
REP02	34	52	2	1	5439	YES	scRNA‐seq
REP03	29	43	2	0	7849	YES	scRNA‐seq
REP04	29	51	1	1	6634	NO	IHC
REP05	33	43	3	1	3858	NO	IHC
REP06	28	50	0	0	24309	YES	IHC
REP07	22	51	0	0	10653	YES	IHC
REP08	40	56	1	0	32160	YES	IHC
REP09	31	54	1	1	8988	YES	IHC
REP10	29	44	1	1	26355	YES	IHC
REP11	36	45	1	0	14688	YES	IHC
REP12	25	51	2	0	8284	YES	IHC
REP13	31	54	2	1	14665	YES	IHC
REP14	35	60	1	1	3300	NO	IHC
REP15	24	44	1	0	5092	NO	IHC
REP16	31	53	1	1	7387	YES	IHC
REP17	23	37	0	0	8936	YES	IHC
REP18	31	45	0	0	7246	YES	IHC
REP19	30	49	1	0	16154	YES	IHC
REP20	32	45	0	0	4097	YES	IHC
REP21	34	41	1	1	3392	NO	IHC
REP22	24	47	0	0	7108	YES	IHC
REP23	31	51	1	0	8201	YES	IHC
REP24	34	42	0	0	9675	YES	IHC
REP25	29	42	1	0	7912	YES	IHC
REP26	34	44	2	0	7902	YES	IHC
REP27	23	41	0	0	4629	YES	IHC
REP28	40	40	1	1	5248	YES	IHC
REP29	33	48	1	0	13591	YES	Explant
REP30	29	49	1	0	36037	YES	Explant
REP31	29	47	0	0	5558	YES	Explant
REP32	37	47	2	1	14079	YES	Explant
REP33	34	54	0	0	3484	NO	Explant
REP34	34	56	1	0	7489	YES	Explant
REP35	44	45	2	1	4110	YES	Explant
REP36	35	43	1	1	13080	YES	Explant
REP37	39	57	1	1	13631	YES	Explant
REP38	31	48	1	0	3450	YES	Explant
REP39	35	40	2	2	4812	YES	Explant
REP40	19	44	1	0	8534	NO	Explant
REP41	32	53	3	1	3761	NO	Explant
REP42	31	43	0	0	15978	YES	Explant
REP43	33	43	1	0	4125	NO	Explant
REP44	39	45	2	1	5724	NO	Explant
REP45	40	42	2	1	7262	YES	Explant
REP46	32	43	1	0	6119	YES	Explant
REP47	35	56	0	0	7656	YES	Explant
REP48	30	42	0	0	3876	YES	Explant
REP49	33	54	1	0	3898	YES	IF
REP50	36	45	2	2	14688	YES	IF
REP51	36	46	1	1	5966	YES	IF
REP52	29	42	1	0	4819	NO	IF
REP53	38	40	1	0	3210	YES	IF
REP54	41	45	2	1	4446	YES	IF
REP55	42	51	2	1	14829	YES	IF
REP56	34	42	1	0	7802	YES	IF
REP57	36	41	1	1	7628	YES	IF
REP58	25	40	1	0	5793	YES	IF
REP59	30	42	1	0	4560	YES	IF

Samples	Age (years)	Gestational age (days)	Gravidity	Parity	HCG (IU/L)	Viable fetus	Method
IP01	33	42	1	1	21896	YES	scRNA‐seq
IP02	34	42	2	1	31086	YES	scRNA‐seq
IP03	31	54	2	2	37569	YES	Explant
IP04	36	47	2	1	8037	YES	Explant
IP05	37	45	2	2	14765	YES	Explant
IP06	40	42	1	0	10259	YES	Explant
IP07	28	54	0	0	35698	YES	Explant
IP08	32	44	1	1	23675	YES	Explant
IP09	31	54	2	2	37569	YES	Explant
IP10	36	47	3	2	8037	YES	Explant
IP11	37	45	5	2	14765	YES	Explant
IP12	40	42	5	2	10259	YES	Explant
IP13	28	54	0	0	35698	YES	Explant
IP14	32	44	2	1	23675	YES	Explant

Abbreviations: IF, immunofluorescence; HCG, human chorionic gonadotropin; IHC, immunohistochemistry; IP, intrauterine pregnancy, AEP, abortive tubal ectopic pregnancy; REP, ruptured tubal ectopic pregnancy; scRNA‐seq, single cell RNA sequence.

Samples collected by surgeries were processed immediately, including fixation in 10% formalin for histological and immunological analyses, immersed in Tissue Storage Solution (130‐100‐008, Miltenyi) for following scRNA‐seq or flow cytometry staining and in RNAlater (AM7021, Thermo fisher) for following real‐time PCR analyses, and cultured in appropriate media for explant experiments. Samples for histological and immunological analyses were obtained by the Department of Pathology, International Peace Maternity and Child Health Hospital (IPMCH), and processed by RecordBio company following standard protocols briefed later.

### 2.2 Isolation of decidual, fallopian tubal and placental cells

Decidual, fallopian tubal and placental tissue was isolated according to a previously published protocol with minor modifications.^14^, ^20^, ^27^ Briefly, the samples were washed in phosphate‐buffered saline (PBS) medium, macroscopically separated by tweezers and scissors. The single‐cell suspension was made after dissociation, digestion, filtration (Falcon 40‐μm cell strainer) and erythrocyte fragmentation. Then, the cell viability assessment was performed before the next step. In details, for decidua and fallopian tube were digested with 20 mL type I collagenase at 1.0 mg/mL in RPIM 1640 medium (C11875500BT, gibco)/10% foetal bovine serum (FBS) (16000‐044, gibco) with gentle shaking at 37 °C for 1 h and 20 mL type I DNase (11284932001, Roche) at 0.1 mg/mL for 5 min in a shaking incubator. For placenta were digested twice for 30 min each at 37°C with 30 mL mix digestive enzyme (15 mL TrypLE (12604021, gibco) and 15 ml ACCUMAX (7921, Stem cell)) with gentle shaking. The digested suspension was filtered through a 100‐μm cell strainer (CSS‐013‐100, BIOFIL) and 40‐μm cell strainer (352340, Corning), then the enzymatic reaction was stopped by adding 10% FBS. The cells were pelleted by centrifugation at 350 × g for 5 min at 4°C. If the red cells were visualized, the red blood cell (RBC) lysis step was performed: 1‐2 ml of RBC lysis buffer (555,899, BD Biosciences) were added and triturated several times, then incubated for 1–2 minutes on ice. The cells were wash by ice‐cold DMEM medium (C11330500BT, gibco)/10% FBS and centrifugated. After discarding the supernatant, the cells resuspended in 100 μL DMEM medium (C11995500BT, gibco). Cells were analysed with a haemocytometer using trypan blue, and which survival rate is generally above 90%. The cell suspension of mother (decidua or fallopian tube) and foetal (villi) are mixed in a ratio of 1–2, and then resuspended to prepare a suitable cell concentration of 700–1200 cells/μL for 10× Genomics Chromium™.

### 2.3 Single‐cell cDNA library preparation and sequencing

This process was performed by Genergy Bio‐Technology Corporation and in accordance with the manufacturer's protocol. Cells were counted and loaded onto the Chromium Controller (10X Genomics) for a target recovery of 8000 single cells. Generation of gel beads in emulsion (GEMs), barcoding, GEM‐RT clean‐up, complementary DNA amplification and library construction were performed. Qubit was used for library quantification before pooling. The final library pool was sequenced on an Illumina Nova6000 platform using 150‐base‐pair paired‐end reads.

### 2.4 scRNA‐seq data analysis

Mapping to GRCh38 human genome, quality control and read counting of Ensemble genes was performed by CellRanger software with default parameter (v6.0). The raw data and processed data were uploaded GEO datasets (GSE207630). Unsupervised clustering was performed with R (Seurat package version 4.0.5). Genes included in the analysis expressed in a minimum of five cells and the total number of expressed cells that expressed >100 and <6000 genes. The cells containing high percentages of mitochondrial genes to total genes, >20%, and haemoglobin genes, >3%, were flited out. A total of 61,119 qualified cells were used for analysis in scRNA‐seq, including 24,031 qualified cells of IPs (*n* = 2), 14,101 qualified cells of AEPs (*n* = 2) and 22,987 qualified cells of REPs (*n* = 3). Then, variation coefficient of genes was calculated with Seurat. Dimensionality reduction of data was performed by using principal component analysis based on the first 2000 highest variable genes. A k‐nearest neighbour graph was constructed from Euclidean distances in the space of the first 10 significant principal components. Louvain modularity optimization algorithm was utilized to cluster the cells in the graph and clustering results were visualized by using Uniform Manifold Approximation and Projection (UMAP) project.

### 2.5 Differential expression and enrichment analysis

The Seurat FindAllMarkers function performed marker genes expression analysis for each cluster using the Wilcoxon rank sum test (adjust *p*‐value <0.05 and fold‐change threshold >1.2). The list of DEGs (differential expressed genes) per cluster then were subjected to functional enrichment analyses using gene ontology (GO) and Kyoto Encyclopedia of Genes and Genomes (KEGG) implemented in the clusterProfiler R Bioconductor package. Significantly enriched GO terms and KEGG pathways were selected by a threshold FDR (adjusted *p*‐value) ≤ 0.05. Function Dotplot from R package ggplot2 (https://www.rdocumentation.org/packages/ggplot2/versions/3.3.5) and function FeaturePlot from R package Seurat (Version 4.0.5) have been used to generate the dot plot and violin plot.

### 2.6 Pseudotime analysis

The present study used two different algorithms to infer the development trajectories: one implemented in R package monocle3 (http://cole-trapnell-lab.github.io/monocle-release/monocle3/)^28^ and developmental lineages and cellular dynamics implemented in RNA velocity (http://velocyto.org/).^29^

### 2.7 Cell–cell communication analysis

Number of significant ligand‐receptor pairs between any pair of two cell clusters was analysed by the method of CellChat.^30^ Meanwhile, other method of CellPhoneDB was used to infer the cell–cell communication mediated by ligand–receptor complexes.^20^

### 2.8 Cell culture

The human trophoblast cell line HTR8‐SVneo was obtained from Dr. PK Lala (University of Western Ontario, London, Ontario, Canada). The cells were cultured in DMEM/F12 containing 10%foetal bovine serum (FBS) with 1% penicillin–streptomycin (P/S) antibodies. HTR8‐SVneo were subsequently cultured in CSF1 (574,804, Biolegend) medium (100 ng/mL) for 24 h, and then were extracted RNA. Human monocytic cell line (THP‐1) was obtained from the cell bank of Chinese Academy of Sciences (Shanghai, China). THP‐1 monocytes were cultured in THP‐1 medium (RPMI 1640 medium, supplemented with 10% FBS and 1% P/S) at 37°C and 5% CO_2_. The THP‐1 monocytes were differentiated to macrophages with 20 ng/mL phorbol‐12‐myristate‐13‐acetate (PMA) (16561‐29‐8, MedChemExpress) for 48 h. The THP‐1 macrophages were subsequently cultured in THP‐1 medium for 24 h. Latterly, the THP‐1 macrophages the stimulated with human CSF1 (20 ng/mL) for 48 h to Macro2 phenotype.

### 2.9 Explant culture

Explants were cultured according to a previously published protocol.^31^ The placenta tissue was dissected out pieces of villus tips (2–3 mm) and explanted into the 96‐well plate precoated with phenol red‐free Matrigel substrate for 37°C 30 min. Then, the explants were incubated in the DMEM/F12 plus 10%FBS with P/S antibodies. Once the placental villi were anchored on the Matrigel (356237, Corning) and grow outward, it was taken as the 0th hour and the differential concentrations of CSF1 and GW2580 (abs813666, Absin) were added to continue to culture for 48 h (refined as 24th and 48th hour). The images of EVT migration from the explants were recorded daily for 0th, 24th and 48th under a light microscope and the extent of migration was measured using ImageJ software. The experiments were repeated at least five times.

### 2.10 Flow cytometry

For the EVT were resuspended in cell staining buffer. For the macrophage, the single cells of the fallopian tube (location of embryo implantation) were resuspended in cell staining buffer (420201, Biolegend). Cells were blocked with Human TruStain FcX Fc receptor blocking solution (422302, Biolegend) and stained with Zombie Aqua Fixable Viability Kit (423102, Biolegend) that eliminated the influence of dead cells. Then, the EVT was stained with PE. Anti‐human HLA‐G antibody (335905, Biolegend). Cells were carried out using a FACS analyser (BD) and analysis after acquisition was conducted by FlowJo software (V10.4).

### 2.11 Wound healing assay

For assessing cell migration in vitro, the wound healing assay was performed. Cells were seeded into 6‐well plate. When the HTR8‐SVneo cells reached 90%–95% confluence, a 200 μL pipette tip was used to scrap the culture plate along the centre axis of the well. Next, the scraped cells were washed twice by DMEM/F12 medium, cultured in medium under differential conditions, and photographed at 0, and 18 h after scratching. Image J software was used to calculate wound healing percentage. The positive control: DMEM/F12 plus 10%FBS with P/S antibodies (positive medium). The negative control: DMEM/F12 plus 2%FBS with P/S antibodies (negative medium). The different concentrations of GW2580 were mixed in the positive medium. While the different concentrations of CSF1 were mixed in the negative medium. photographed at 0 and 18 h after scratching.

### 2.12 Matrigel‐based invasion assay

Cell invasion was detected by the ability of HTR‐8/SVneo cell to cross the 8‐mm pores of polycarbonate membranes (8‐mm pore size, Corning). 1 × 10^5^ cells per well were plated in the upper chamber precoated with Matrigel membrane. The lower chamber was filled with 500 μL DMEM containing 10% FBS, or 500 μL DMEM containing 10% FBS and 100 ng/mL CSF1. Cells in the lower chamber were stained for 30 min with crystal violet after 24 h of incubation. The number of migrated cells was counted using an inverted microscope (Nikon).

### 2.13 Immunofluorescence staining

All the samples of paraffin blocks were collected by the Department of Pathology, IPMCH, followed by cut into 5‐μm slices using the microtome (Leica, RM2235). The sections for immunological analyses were performed by the Double Homologous antibody Fluorescence labelling Kit (RecordBio Biological Technology) and in accordance with the manufacturer's protocol. Co‐staining of three antibodies was performed using a three‐colour Fluorescence kit (Recordbio Biological Technology) based on the tyramide signal amplification (TSA) technology according to the manufacture's instruction. In brief, paraffin sections were deparaffinized, rehydration, antigen retrieval, and followed by blocked with 3% H_2_O_2_ and 2% bovine serum albumin (BSA) in PBS. Subsequently, sections were incubated at 4 overnight with primary antibodies, Room temperature 1 h with secondary antibodies, followed by 10 15 mins with TYR‐488/TYR‐CY3 or TYR‐488/TYR ‐CY3/TYR‐CY5 solution. The primary antibodies including rabbit anti‐HLA‐G (1:200, 79769, Cell Signalling Technology), rabbit anti‐CSF1 (1:100, ab233387, Abcam), rabbit anti‐CSF1R (1:100, ab183316, Abcam), rabbit anti‐MMP2 (1:200, 40,994, Cell Signalling Technology), mouse anti‐Oviductin (1:100, sc‐377267, Santa Cruz Biotechnology), rabbit anti‐MKI67 (1:200, ab16667, Abcam), mouse anti‐ISYNA1(1:100, sc‐271830, Santa Cruz Biotechnology), rabbit anti‐CD80 (1:200, ab225674, Abcam), and mouse anti‐CD206 (1:200, 60143‐1‐Ig, Proteintech).

### 2.14 Immunochemical staining

Paraffin blocks were cut into 5‐μm slices, then deparaffinized, rehydrated and processed for antigen retrieval by a standard microwave heating technique in antigen retrieval solution (pH = 8) (RecordBio, #RC016). Endogenous peroxidase activity was quenched by incubating the sections in 3% H_2_O_2_ for 25 min. The sections were incubated with 0.2% Triton X‐100 and 3% bovine serum albumin (BSA) in PBS for 1 h at room temperature and were then incubated overnight with primary antibodies at 4°C. Primary antibodies used for IHC include: rabbit anti‐HLA‐G (1:200, 79,769, Cell signalling technology), rabbit anti‐MMP2 (1:200, 40,994, Cell signalling technology), rabbit anti‐CSF1 (1:200, ab233387, Abcam), rabbit anti‐CSF1R (1:100, ab183316, Abcam) and rabbit anti‐PAX8 (1:1000, 10336‐1‐AP, Proteintech). After overnight, samples were washed in PBS and incubated with the secondary antibody. Slides were visualized using diaminobenzidine tetrahydrochloride (RecordBio), and nuclei were counterstained with haematoxylin.

### 2.15 Quantitative real‐time PCR analysis

Total RNA of each sample was isolated by RNA Isolator Total RNA extraction Reagent (R401‐01, Vazyme). The cDNA was synthesized with the HiScript II Q RT SuperMix (R223‐01, Vazyme) according to manufacturer's instructions, and real‐time PCR reaction was done with QuantStudio 7 Flex Real‐Time PCR system (Life Technologies) and ChamQ Universal SYBR qPCR Master Mix (Q711‐02, Vazyme). The amount of target mRNA (relative quantity, RQ) was determined using the ∆∆Ct method with GAPDH as the internal control with three replications. GAPDH‐F: GTCTCCTCTGACTTCAACAGCG. GAPDH‐R: CCACCCTGTTGCTGTAGCCAA. MMP2‐F: GATACCCCTTTGACGGTAAGGA. MMP2‐R: CCTTCTCCCAAGGTCCATAGC.

### 2.16 Statistical analyses

For continuous variables, Student's *t*‐test was used if the data were normally distributed and have equal variances, and Welch's *t* test was applied if the data were normally distributed and have unequal variances. One‐way ANOVA was applied to examine the differences of baseline characteristics among the three groups (IP, AEP, REP). Continuous variables are presented as mean ± SD/standard error of mean (SEM). The linear regressions of measured parameters were calculated with ages and gestational days as independent variables. Invasion depth of EVT in EP refers to the differentiated EVT close to cytotrophoblast cells column to the distance between the adjacent serous layer of EVT. Statistical analyses were performed using GraphPad Prism 8 (GraphPad Software Inc., La Jolla, CA, USA). *p*‐Values < 0.05 were considered statistically significant.

## 3 RESULTS

### 3.1 EVTs are more abundant and show deeper invasion in REP than AEP

**FIGURE 1:**
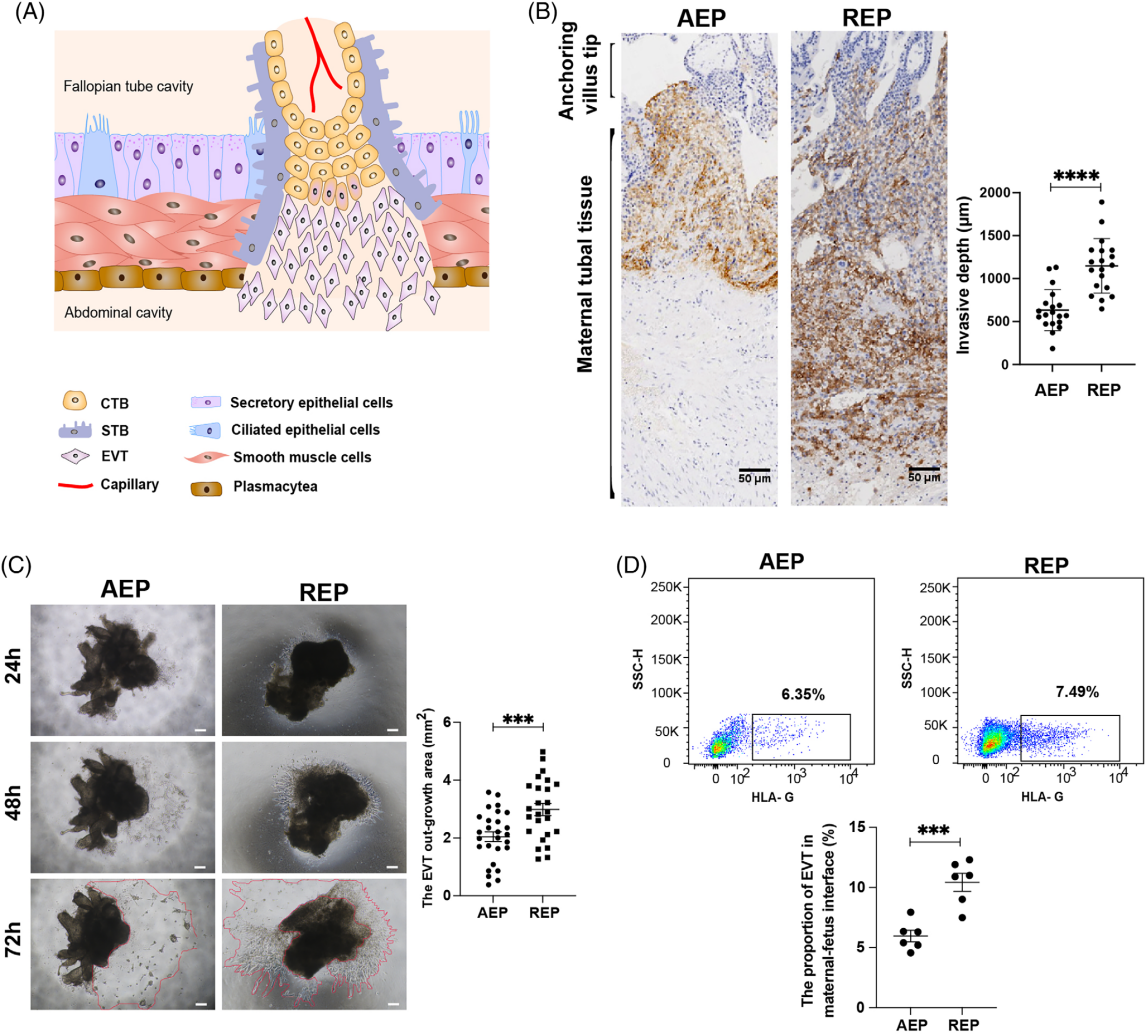
The EVT exhibits a larger amount and deeper invasion in REP. (A) Diagram illustrating a placental villus at the maternal–foetal interface in the first trimester of ruptured tubal ectopic pregnancy showing the different trophoblast subsets (STB, CTB, and EVT) and cell types of oviducts (secretory epithelial cells, ciliated epithelial cells, smooth muscle cells, and plasmacytea). CTB, cytotrophoblast; EVT, extravillous trophoblast; STB, syncytiotrophoblast. (B) Immunohistochemical staining for HLA‐G in maternal–foetal interface sections, which shows the invasion depth of EVT (representative images from AEP =18, REP = 19). The statistical analysis demonstrates the invasion depth of EVT between AEP and REP. Scale bars, 50 μm. Data are represented as mean ± SEM. (C) Phase‐contrast images of villus explants cultured for 24, 48, and 72 h. Representative images from AEP = 13, REP = 20. Scale bars, 200 μm. The EVT growth area in the REP group was significantly larger than that in the AEP group after 72 h. Data are represented as mean ± SEM. (D) FACS gating strategy to analyse the proportions of EVT between AEP (*n* = 6) and REP (*n* = 6). Data are represented as mean ± SEM. AEP, abortive tubal ectopic pregnancy; REP, ruptured tubal ectopic pregnancy. **p < 0.01; ***p < 0.001; ****p<0.0001.

Since the placenta is implanted in the fallopian tube wall at different invasion depths in REP and AEP, we speculated that, in REP, EVT exhibit a high proliferative capacity and invade the serosal layer to ensure an adequate blood and nutrient supply to support embryo development, but which together result in tubal rupture (Figure 1A). Immunostaining for HLA‐G, an EVT marker, revealed that the trophoblast invasion was deeper and broader at the maternal–foetal interface in REP than in AEP (REP: 1148.07 ± 317.36 μm vs. AEP: 632.10 ± 238.75 μm) (Figure 1B) and that the EVT exhibited more aggressive invasion and proliferation (Figure 1C). Furthermore, flow cytometry using the HLA‐G marker demonstrated that placental villi from the REP group appeared to have a markedly higher abundance of EVT at the maternal–foetal interface than those of the AEP group (Figure 1D). These results implied that the excessive invasion by EVTs in the narrow space of the fallopian tube contributes to the development of REP and tubal rupture.

### 3.2 EVT populations are overrepresented in REP compared to AEP and IP

**FIGURE 2:**
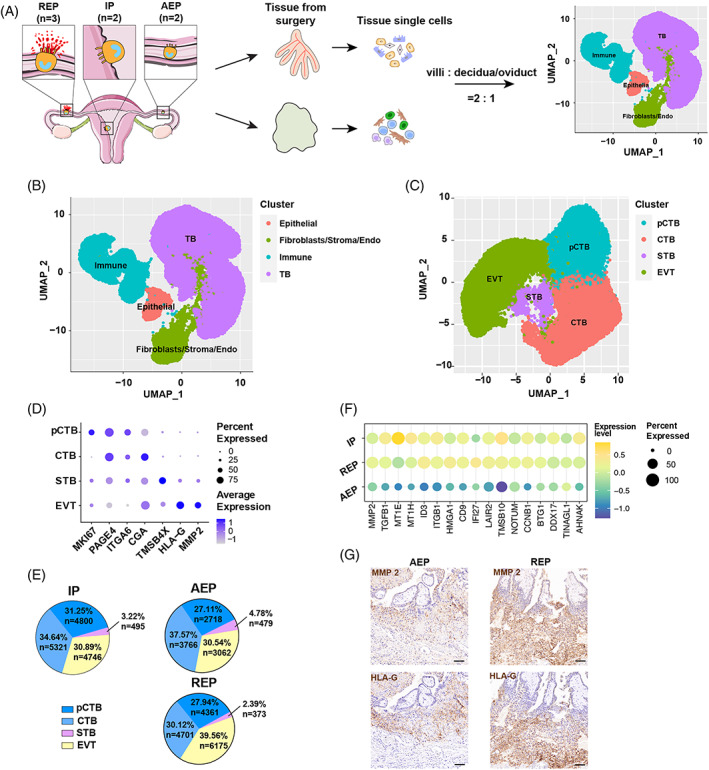
The differences in proportion and transcriptome of EVT between AEP and REP. (A) Workflow for single‐cell transcriptome profiling of fallopian tube and placenta. Numbers in parentheses indicate the number of individuals analysed. AEP (*n* = 2), REP (*n* = 3), and IP (*n* = 2). (B) Placental and fallopian tubal cell clusters from 10× Genomics scRNA‐seq analysis visualized by UMAP. Colours indicate cluster. TB, trophoblast cell. Immune, immune cells. Epithelial, Epithelial cells. Fibroblasts/Endo, Fibroblastic cells, stromal cells (IP), or Endothelial cells. (C) UMAP visualization of trophoblast cell analysis. Each point represents one cell that is coloured by its cell type as shown in the legend. CTB, cytotrophoblast, EVT, extravillous trophoblast; p, proliferative; STB, syncytiotrophoblast. (D) The expression level of each cluster‐defining markers and percentage of positive cells in each cluster. (E) Comparison of each trophoblastic type of number and proportion in three groups (IP, AEP and REP). (F) Expression of genes related to invasion or epithelial‐mesenchymal transition in trophoblast cells in three groups (IP, AEP and REP). AEP, abortive tubal ectopic pregnancy. IP, intrauterine pregnancy; REP, ruptured tubal ectopic pregnancy. (G) Immunochemical staining for HLA‐G (EVTs marker) and MMP2 (invasive marker) (representative images from AEP = 3, REP = 3). Scale bars, 100 μm.

To systematically investigate differences in the respective REP, AEP and IP implantation microenvironments by identifying EVT subtypes and characterizing intercellular communications specific to REP, maternal and placental tissues were collected from the maternal–foetal interface of each pregnancy type (IP, AEP and REP) and isolated cells were mixed for single‐cell sequencing (Figure 2A). The cell populations detected in IP patients showed extensive overlap in cell identities and cluster distributions, aligning well with another recent study^20^ and validating the reliability of our results (Figure S1A). The four major cell types separately clustered in UMAP analysis were then annotated as trophoblast cells, immune cells, epithelial cells, or fibroblasts/stroma/endothelial cells, based on their cluster‐specific expression of marker genes (Figure 2B, Figure S1B). Subsequent trophoblast lineage reconstruction identified four trophoblast types: proliferative/stem cytotrophoblasts (pCTBs), cytotrophoblasts (CTBs), syncytiotrophoblasts (STBs) and extravillous trophoblasts (EVTs) (Figure 2C,D). By contrast, EVTs were obviously overrepresented in the REP group (Figure 2E) and showed aberrantly high expression of the invasion‐related genes (MMP2, LAIR2, NOTUM and others) (Figure 2F) compared with that in AEP patients at similar gestational ages. Furthermore, the immunochemistry of EVTs (HLA‐G+) collected from the implantation site demonstrates that they expressed higher levels of MMP2 in REP compared with that in AEP patients (Figure 2G).

### 3.3 Preferential differentiation into EVT3 subset with higher invasive gene expression in REP

**FIGURE 3:**
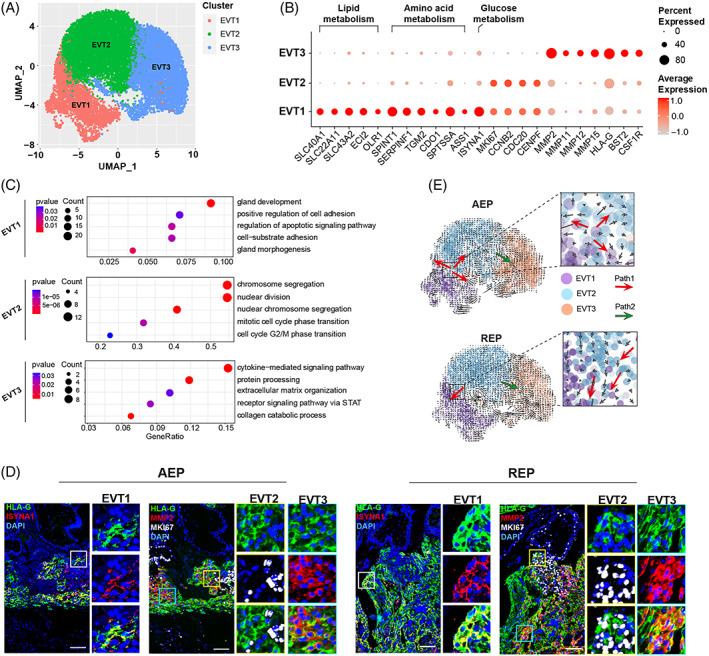
Aberrant differentiation trajectory of EVT impairs tubal integrity in REP. (A) UMAP plots of EVT subsets analysis. Further EVT subgroup analysis classified them into three subsets (EVT1, EVT2, EVT3). (B) Visualization of each subset‐defining marker using bubble plots, with shades of colour representing expression level and sizes of dots representing the percentage of positive cells. (C) GO analysis of enriched terms across upregulated genes of EVT subsets. The terms with *p* < 0.05 are selectively shown. (D) Immunofluorescence for HLA‐G (EVTs marker, green), ISYNA1 (EVT1 marker, red), MKI67 (EVT2 marker, white), and MMP2 (EVT3 marker, red) (representative images from AEP =11, REP = 11). Scale bars, 100 μm. (E) Velocity analysis of UMAP plots, colours represent different EVT subsets, arrows indicate the projection of the RNA velocity field in this image, and the direction and size indicate the average velocity vector predicted on the unit network. AEP, abortive tubal ectopic pregnancy; EVT, extravillous trophoblast; IP, intrauterine pregnancy; REP, ruptured tubal ectopic pregnancy.

Further examination of marker expression revealed three distinct subsets within the EVT cluster, including EVT1 (ISYNA1+, SPINT1+), EVT2 (MKI67+, CCNB2+) and EVT3 (MMP2+) (Figure 3A). EVT1 cells displayed characteristic upregulation of metabolism‐related genes (Figure 3B) and were enriched in gland development and cell adhesion (Figure 3C, Table S1), reflecting their enhanced function in obtaining maternal nutrients. EVT2 was defined by a relatively high expression of MKI67 and CCNB2 (Figure 3B **)**, which are associated with cell cycle and cell division (Figure 3C, Table S1 **)**, suggesting that this cell subtype could function in determining proliferative potential. GO analysis further showed enrichment in feature genes related to extracellular matrix (ECM) organization and positive regulation of EVT invasion in EVT3 cells (Figure 3B, C, Table S1).

To validate the existence of three cell subtypes, we performed immunohistochemistry (IHC) staining with anti‐ISYNA1, anti‐MKI67 and anti‐MMP2 antibodies in serial sections of human placental villi embedded in paraffin. Our results revealed that ISYNA1+ EVTs exhibited little or low expression of HLA‐G at the proximal site of the fallopian tube secretory epithelial cells (FTSECs), while MKI67+ EVTs were localized at the distal end of the column, with invasive EVTs (MMP2+) showing dispersed localization in the fallopian tube (Figure 3B,D **)**. Notably, invasive genes were expressed at a higher level in the EVT3 cells of REP patients than in the same subset of AEP patients (Figure S2A).

Trajectory analysis with Monocle3 and RNA velocity analysis of EVT subpopulations were consistent in REP samples, showing two distinct EVT lineages in which EVT2 cells could differentiate into either EVT1, with absorbing nutrients to support foetal growth (path 1), or into EVT3, with a high invasive capacity to break into the fallopian tubal wall (path 2) (Figures 3Band S2B). In addition, in the AEP group, EVT2 cells showed largely aberrant differentiation into the EVT1 subset, and EVT3 cells with weaker invasive abilities (Figure 3E, Figure S2A, B), implying that maternal nutrient absorption and EVT invasion could be insufficient in these patients, possibly leading to miscarriage.

### 3.4 Macrophage subsets differ between AEP and REP patients

**FIGURE 4:**
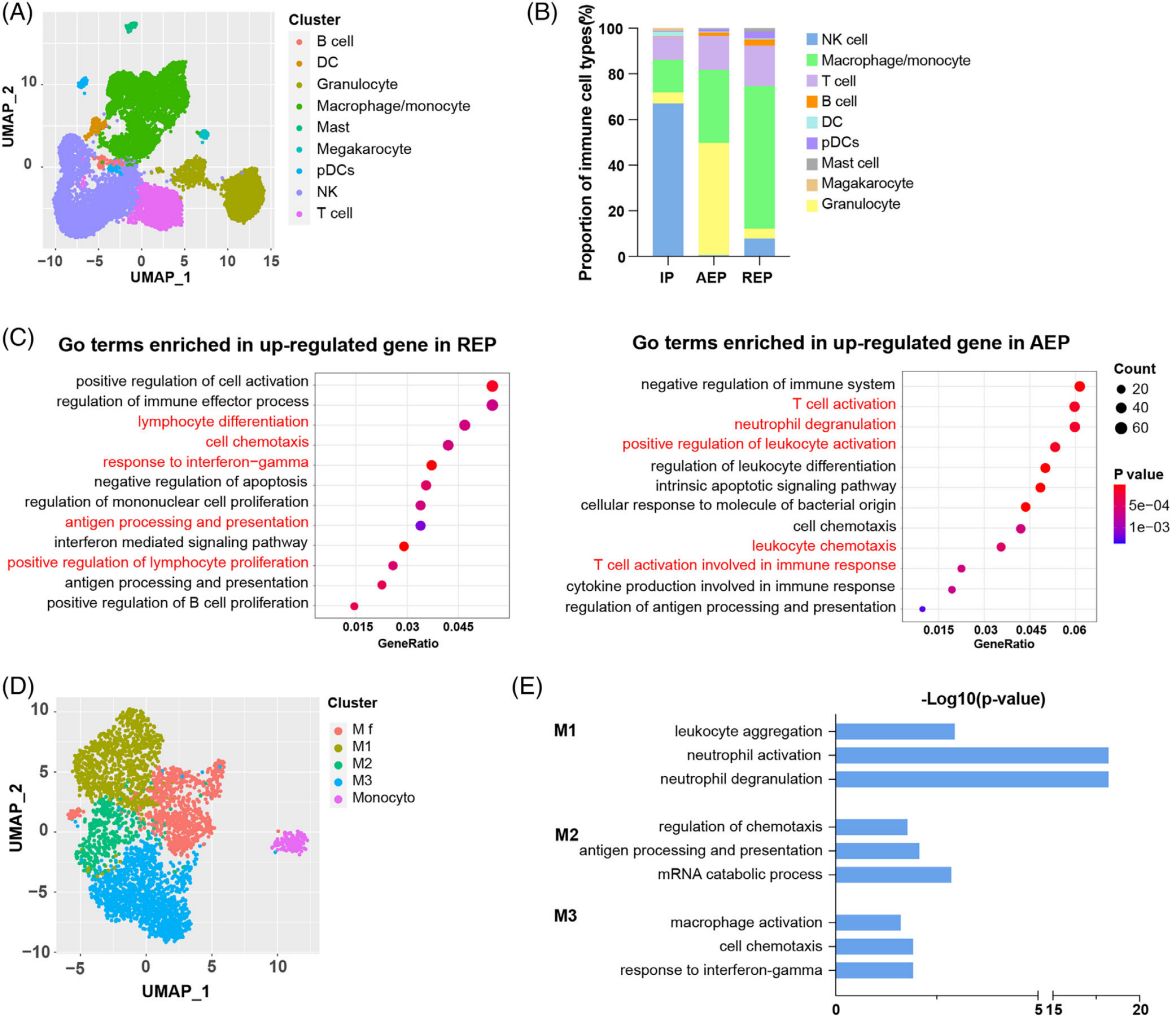
Change in proportions of macrophage between AEP and REP patients. (A) UMAP visualization of immune cluster obtained from Figure 2B. Cells are coloured by the identified clusters. DC, dendritic cells; NK, and natural killer cells; pDCs, Plasmacytoid dendritic cells. (B) Comparison of each immune cell proportion in three groups (IP, AEP and REP). (C) GO analysis of enriched terms across upregulated and downregulated genes of macrophage cells. Terms with *p* < 0.05 are selectively shown. (D) UMAP plots of macrophage subtypes. Each dot denotes one cell which is coloured by the identified subtypes. f, fetus; M, macrophage. (E) GO analysis of enriched terms across upregulated and downregulated genes of macrophage. AEP, abortive tubal ectopic pregnancy; IP, intrauterine pregnancy; REP, ruptured tubal ectopic pregnancy.

The implantation immune microenvironment is a complex, multilevel interaction network that provides major contributions to regulating EVT function. Identification of immune‐related cells using known marker genes revealed that macrophages/monocytes were the dominant cell type in both AEP and REP, while NK cells accounted for a relatively small population (Figures 4A and S3A).^32^, ^33^, ^34^ Moreover, the proportion of macrophages/monocytes was higher in REP patients than in AEP (Figures 4B and S3B‐3C). Volcano plots of differentially expressed genes (DEGs) in macrophages (Figure S3D) and GO analysis of up‐regulated DEGs genes between the REP and AEP groups (Figure 4C) demonstrated that macrophages in REP patients were mainly involved in lymphocyte differentiation, cell chemotaxis and antigen processing and presentation, while macrophage DEGs in AEP patients were mainly enriched in T‐cell activation, neutrophil degranulation and positive regulation of leukocyte activation (Figure 4C).

Further clustering analysis and annotation with marker genes identified four macrophage subtypes and monocytes (Figures 4D and S3E–H), including foetal macrophage (Mf) cells with high expression of C1QA and PAGE4,^20^ an M1 subset with high S100A8/S100A9 expression, similar to the Macro1 subset reported in IP,^35^ and an M2/3 subset with high CD206 expression similar to the alternatively activated phenotype (Macro2) subset in the IP^34^ (Figures 4D S3G, H). Based on scRNA data obtained from normal fallopian tubes (ampullar), this subcluster of macrophages was only found in the fallopian tube of a tubal pregnancy (including both REP and AEP) (Figure S3H, I),^36^ suggesting that the presence of macrophage subsets in TEP was related to pregnancy (Figure S3H, I). These overall observations suggested that the M1 subgroup, which was dominant in AEP, could most likely promote an inflammatory response and inhibit trophoblast invasion (Figure 4E, Figure S3E, F, Table S2). In contrast, the M2/3 subgroups, dominant in REP patients, potentially functioned in maintaining the microenvironment balance and conceptus (Figure 4E, Figure S3E, F, Table S2). At the maternal–foetal interface in EP patients, high expression of anti‐inflammatory factors, such as M‐CSF (CSF1), IL4 and IL10 could promote the monocyte tendency to differentiate into anti‐inflammatory phenotype macrophages (Macro2) and reinforce the capacity for EVT invasion.^33^, ^37^ These results showing enrichment with macrophages in REP could explain the enhanced EVT invasion behaviour and imbalance in the immune microenvironment of the maternal–foetal interface.

### 3.5 Accumulation of M2 macrophages and EVT invasion induced by a CSF1/CSF1R regulatory axis in FTSECs

**FIGURE 5:**
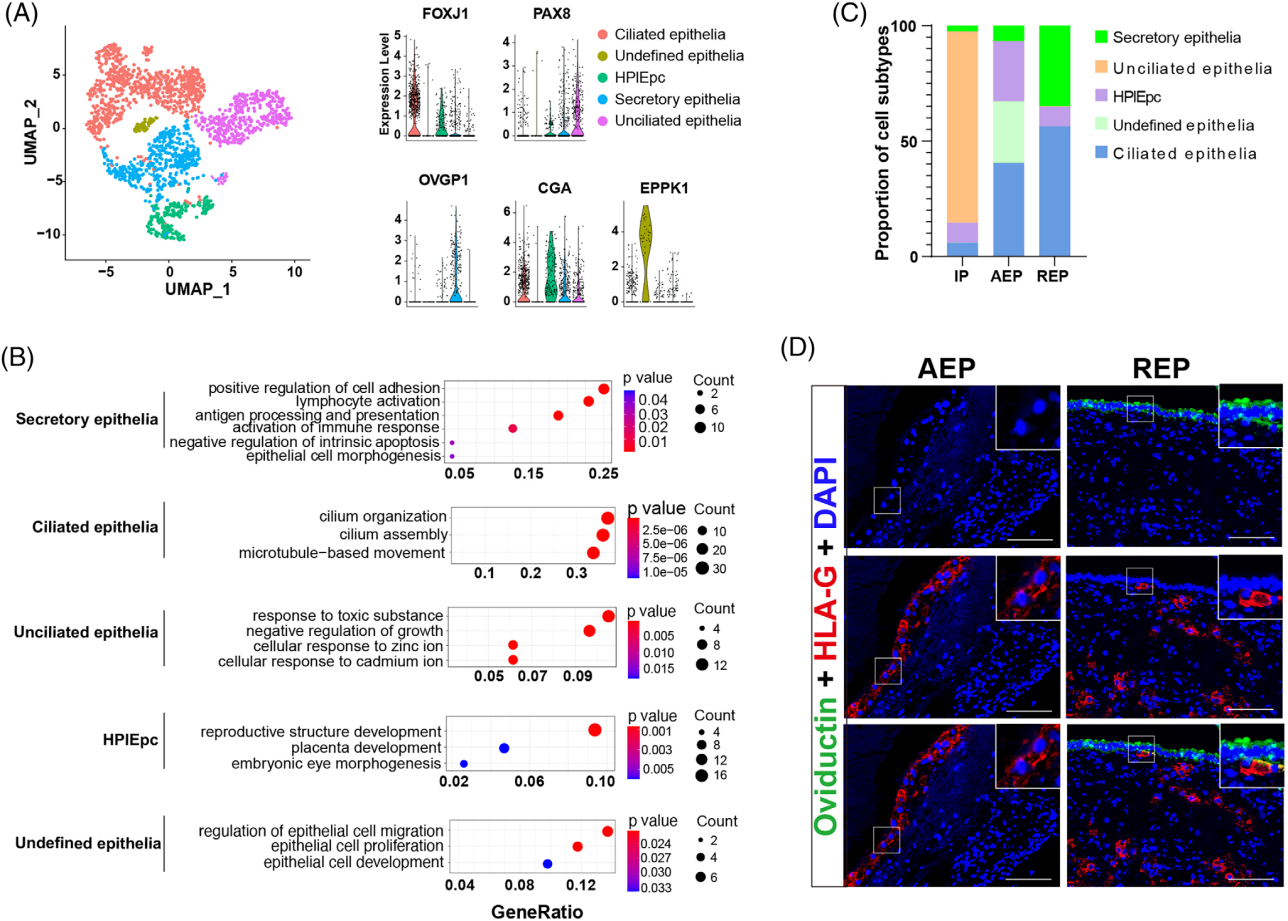
REP patients retain almost complete secretory epithelial cells. (A) Subpopulation analysis of epithelial cells UMAP diagram. Colours represent different subpopulations. HPlEpc, human placental epithelial cells, Violin plots of marker genes of each cell subpopulation. (B) Functional annotation of each epithelial cell subpopulation. (C) Cell proportions of epithelial cell subpopulations in REP, AEP, and IP groups. Colours represent identified subtypes. (D) Immunofluorescence for HLA‐G (EVT marker) and OVGP1 (secretory epithelial cells marker) in the maternal‐foetal interface of AEP and REP samples (representative images from AEP = 8, REP = 8). Green represents OVGP1, red represents HLA‐G, and blue represents DAPI (nucleus marker). Scale bars, 100 μm. AEP, abortive tubal ectopic pregnancy; IP, intrauterine pregnancy; REP, ruptured tubal ectopic pregnancy.

To explore the association between maternal epithelia and EVT, we next identified epithelial cells based on their transcriptomic profile. Notably, epithelial cells (EPCAM+) could be partitioned into five cell types, including ciliated epithelia (FOXJ1+), unciliated epithelia (PAX8+), secretory epithelia (PAX8+, OVGP1+), human placental epithelial cells (HPlEpC, CGA+) and undefined epithelia (EPPK1+) (Figure 5A). The latter of these subtypes, EPPK1+ undefined epithelia, was specific to AEP patients and enriched with feature genes related to phenotypes, such as epithelial proliferation, migration and development (Figure 5B, Table S3). Ciliated cells were enriched with genes related to normal tubal cilia function and the HPlEpC subset was comprised of foetal epithelial cells (Figure 5B, Table S3). Among these clusters, FTSECs showed enrichment for feature genes involved in immune response and lymphocyte activation, as well as cell adhesion, suggesting that these cells participated in EVT invasion (Figure 5B, Table S3). FTSECs accounted for more than 30% of all epithelial cells in REP, but only 6.7% in AEP samples (Figure 5C, Figure S4A). In light of the remarkably high proportion of these cells, we performed immunostaining for OVGP1, a secretory epithelial marker and HLA‐G, an EVT marker, revealed that FTSEC populations were smaller in AEP patients compared with REP patients and EVT was located under the intact FTSECs in REP (Figure 5D).

Aside from epithelial cells, the fibroblast/stroma/endothelia cells were also identified in nine cell types (Figure S4B‐4C). The F3/F4 cells were dominant in the AEP group, and DEGs enriched in this population were largely related to neutrophil activation (Figure S4D), suggesting that the F3/F4 subsets were likely responsible for actively promoting granulocyte accumulation in AEP patients (Figure S4D). Collectively, these results implied that cytokines and chemokines secreted from FTSECs could interact with macrophages and EVTs at the maternal–foetal interface, stimulating macrophage proliferation and potentially modulating EVT function via transcriptomic reprogramming.

**FIGURE 6:**
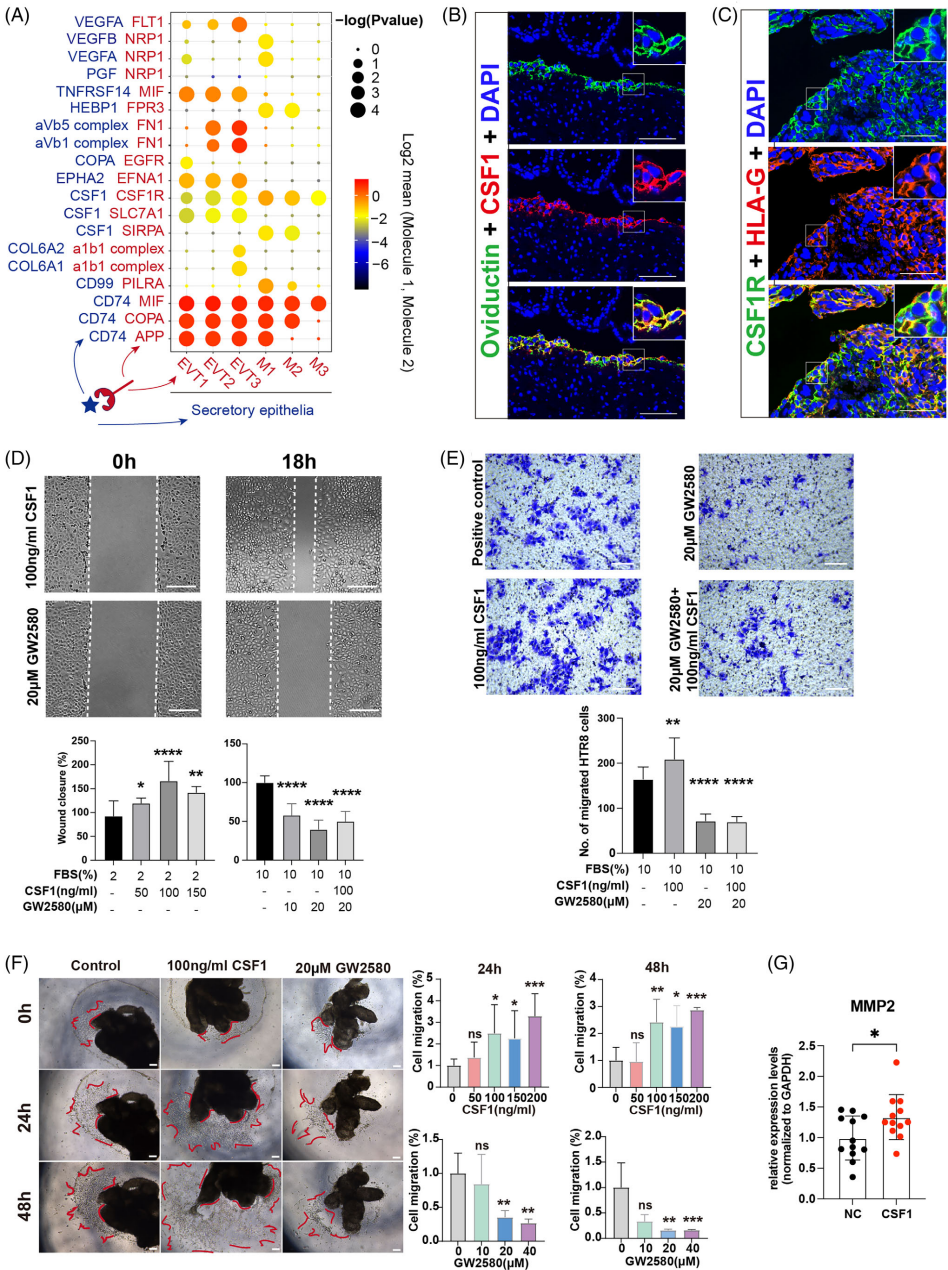
Secretory epithelial cells for macrophage accumulation and EVTs invasion by CSF1‐CSF1R regulatory axe. (A) Dot plot of predicted ligand‐receptor interactions. The circle size represents the p‐value, and the colour is the gene level indicating the interaction. (B) Immunofluorescence for OVGP1 (secretory epithelial cells marker) and CSF1 in the maternal‐foetal interface of REP. Green represents OVGP1, red represents CSF1, and blue represents DAPI (nucleus marker). Scale bars, 100 μm. (C) Immunofluorescence for HLA‐G (EVTs marker) and CSF1R in the maternal‐foetal interface of REP. Green represents CSF1R, red represents HLA‐G, and blue represents DAPI (nucleus marker). Scale bars, 100 μm. (D) Representative images showed the migration of HTR8/SVneo cells in the differential treatments (0 h, 18 h). The migration of HTR8/SVneo cells was compared between 0 and 18 h. Scale bars, 50 μm. Data are represented as mean ± SD. (E) Representative images of invaded HTR8/SVneo cells treated with different concentrations of CSF1 or GW2580. Scale bars = 100 μm. The number of invaded cells was compared in different groups. Data are represented as mean ± SD. (F) The EVT growth area of the explants was recorded by microscopy after 24 and 48 h, respectively. Scale bars, 200 μm. The EVT growth area in the CSF1 group was significantly larger than that in the GW2580 group after 24 and 48 h. Data are represented as mean ± SEM. (G) Real‐time qPCR analysis of MMP2 expression in HTR8/SVneo cells treated with CSF1 compared to control. Data are represented as mean ± SD. AEP, abortive tubal ectopic pregnancy; IP, intrauterine pregnancy; REP, ruptured tubal ectopic pregnancy. . *p < 0.05, **p < 0.01. ***p < 0.001. ****p < 0.0001.

CellChat analysis was conducted to explore this possibility, which identified FTSECs as the predominant intercellular communication “hub,” with secreted signals from these cells predicted to interact with both macrophages and EVTs (Figure S5A). To further investigate the mechanism of cell‐to‐cell communication, we applied CellPhoneDB to identify interaction pairs that were enriched in EP (Figure 6A). In IP, the colony‐stimulating factor‐1 (CSF1) is synthesized in high concentrations by the endometrium during pregnancy and targeted to CSF1R of trophoblast.^38^, ^39^ Many studies have revealed that CSF1 regulates the proliferation, differentiation and survival of macrophages,^40^ regulating placental development^41^ and supporting EVT proliferation and migration.^42^ These listed functions are accompanied by substantial enrichment of macrophages at the implantation site compared with their distribution throughout the rest of the fallopian tube.^43^ In addition, cell‐to‐cell communication analyses in previous studies showed that the CSF1‐CSF1R pair links the dNK1 subset with EVTs in healthy decidua of IP patients,^20^, ^44^ but forms a connection between FTSECs and macrophages in EP patients.^45^, ^46^ Furthermore, immunofluorescent co‐staining for OVGP1 and CSF1 confirmed that CSF1+ FTSECs were abundant at the implantation site in REP patients (Figure 6B), while EVTs enriched at the implantation site expressed both HLA‐G and CSF1R, implying that they could interact with FTSECs (Figure 6C). In addition, FTSECs in REP samples displayed higher CSF1 expression than in AEP samples (Figure S5B, C), while its receptor, CSF1R, was expressed at similar levels by EVTs in both pregnancy types (Figure S5D). Moreover, the FTSECs in AEP were enriched with genes (BID, EIF5A and HIF1A) related to apoptotic signalling (Figure S5B).^47^, ^48^, ^49^ FTSECs were almost completely absent at the fallopian tube‐trophoblast interface in AEP patients, while those that were detected expressed CSF1 at low levels (Figure 5D, Figure S5B, C). The overexpression of CSF1 in the FTSECs of REP suggested a possible role in regulating EVT proliferation and invasion.

To investigate whether the CSF1/CSF1R ligand‐receptor pair indeed played a role in promoting EVT proliferation and invasion, we conducted functional assays using HTR8‐SVneo cells and villous explant cultures. Previous studies showed that upregulation of CSF1 expression can induce a dose‐dependent increase in trophoblast proliferation.^42^ To test this possibility, we applied GW2580, a CSF1R antagonist that blocks CSF1‐induced proliferation, to cells in in vitro wound closure assays **(**Figure 6D **)**, transwell assay **(**Figure 6E **)** and villous explant cultures **(**Figure 6F **)**. The results indicated that exogenous CSF1 could obviously stimulate the migration and invasion of HTR8‐SVneo, while exposure to GW2580 inhibited their migration and invasion (Figure 6D,F, Figure S5E). QPCR‐based relative expression assays indicated that stimulation with exogenous CSF1 (100 ng/mL) significantly increased MMP2 expression in the HTR8‐SVneo (Figure 6G). These cumulative results indicated that high CSF1 levels could likely increase MMP2 expression, thereby promoting EVT invasion. In addition, after inducing THP‐1 monocytes to differentiate into macrophages, culturing these macrophages in a medium containing purified CSF1 resulted in their polarization towards a *CD206* + *CD80*‐ Macro2‐like phenotype (Figure S5F). Altogether, these experiments demonstrated that the aberrant upregulation of the CSF1/CSF1R ligand‐receptor pair between FTSECs and EVTs/macrophages in REP appears to drive the expansion of EVTs and macrophages, promoting EVT invasion and ultimately resulting in REP.

## 4 DISCUSSION

Until recently, the fallopian tube, which is an essential component of normal reproductive function in transporting the oocyte or embryo to the uterus, has received relatively little research attention. As a result, little is known about how tubal epithelial cells could affect trophoblast cells after implantation. In IP, endometrial stromal cells transform into decidua stromal cells by mesenchymal‐epithelial transition during decidualization, playing vital roles in regulating trophoblast (EVT) differentiation and function, which impacts pregnancy outcomes.^34^, ^50^ The decidua spongiosa contain hyper‐secretory glands which provide histotrophic nutrition to support early embryonic growth.^20^ However, the pathological factors that lead to tubal rupture in the fallopian tube epithelium without decidualization in EP patients remain unclear. In this study, which focused on the maternal–foetal interface in patients with REP or AEP, the EVTs were shown to invade in larger numbers and deeper into the tubal serosal layer in REP than in AEP. Using scRNA‐seq, we constructed a comprehensive single‐cell transcriptomics atlas of the maternal–foetal interface in both REP and AEP, which identified previously unrecognized EVT subtypes and provided a robust differentiation trajectory, especially focusing on the mechanism leading to tubal rupture. These cumulative results revealed that CSF1 secretion by FTSECs promotes EVT invasion and induces macrophage polarization to the macro2 response through interactions with CSF1R.

Several studies have illustrated the different EVT subtypes that participate in IP. Based on their distinct spatial location, EVTs can be subdivided into two major subtypes: interstitial EVT cells (iEVT, which invade the decidualized endometrium), and endovascular EVT cells (which invade and remodel maternal spiral arteries).^51^ Alternatively, EVTs can be functionally categorized as either proliferative EVTs (pEVT) or invasive EVTs.^20^ Other recent scRNA‐seq studies have identified yet more subtypes of EVT in IP patients.^27^, ^52^ By combining these previously described spatial or functional EVT subtypes with the marker genes for EVT populations in IP patients, we identified three EVT subtypes in AEP and REP, including EVT1 (nutrition), EVT2 (proliferation) and EVT3 (invasion). The dynamic changes in HLA‐G expression in EVTs are similar to those of MMP2. Moreover, the EVT3 subset displays the highest HLA‐G expression, revealing that the EVT3 subset participates in the invasion process and maternal‐foetal immune tolerance in an imbalanced state.^53^ Furthermore, compared with the AEP group, pseudotime trajectory analysis showed that EVT2 cells in REP can transition to EVT1 (path 1), which actively transport nutrition to support villous development, or EVT3 (path 2) cells, which are responsible for the excessive invasion of the tubal wall after placental implantation.

CSF1 encodes a cytokine that positively regulates the production, differentiation and function of macrophages, and which is therefore likely involved in placental development^41^ and promoting EVT proliferation and migration.^42^ Compared with AEP, REP has FTSECs with more abundant and higher CSF1 expression. Cellchat analysis implied that CSF1+ FTSECs can interact with EVTs and macrophages. This interaction was experimentally supported by our findings that CSF1 binding with CSF1R on EVTs stably overexpressing MMP2 promotes EVT proliferation, invasion and survival in vitro. The results in this study also show that FTSECs express VEGF‐A/B, which binds to receptors on EVTs (especially EVT3), subsequently enhancing EVT invasion.^54^ These results suggest that further mechanistic studies are needed to determine how VEGFs function in modulating EVT3 invasion. Moreover, the initiation of tubal rupture is a complex process that requires contributions from several EVT subpopulations, thus warranting further experimental examination.

In the normal fallopian tube, in the nonpregnant state, the immune landscape is dominated by T cells (especially CD8+ T cells),^36^, ^55^ whereas in EP, macrophages are the most abundant leukocyte population within the human fallopian tube,^32^ which is consistent with our results in the present study. Hence, ectopic implantation can influence the proportions of different macrophage populations compared with their relative abundance in nonpregnant women. In AEP patients, the M1 subgroup is the dominant population, exhibiting a Macro1‐like phenotype characterized by production of *TNF‐α* and *iNOS*, which induces apoptosis in trophoblasts and inhibits EVT invasion, ultimately leading to abortion of the ectopic embryos.^21^, ^22^ By contrast, the M2/M3 subgroups are dominant in REP and exhibit macro2‐like functions, possibly playing important roles in immune tolerance, placental vascularization and EVT invasion.^33^, ^56^ In EP, fallopian tube epithelial cells could be involved in modulating the number and function of resident immune cells.^34^ In addition, our results suggest that CSF1 expression can induce an anti‐inflammatory macro2‐like phenotype, which is consistent with findings reported in previous studies,^40^, ^57^ further highlighting the capacity for invasion in EVTs. Collectively, CSF1+ FTSECs induce monocyte polarization to the macro2 phenotype, and changes in the proportions and subtypes of these cells (FTSECs and macrophages) in REP supports the likelihood that EVT dysfunction could contribute to tubal rupture.

While three EVT subsets are identified in EP, the mechanisms regulating the EVT subtype trajectories remain unexplored. In the present study, HTR8‐SVneo and villous explants were applied to verify the role of the CSF1/CSF1R ligand‐receptor pair in EVT proliferation and invasion. However, our results should be verified in a wider range of trophoblast cell lines or trophoblast organoids in future studies.

In addition, the fallopian tube samples were collected after implantation and may not explain the aetiology responsible for differences in FTSECs between AEP and REP.

In summary, our cell type‐specific data provide new insights into the aetiology of tubal rupture of REP patients by identifying key interactions between CSF1+ FTSECs and their surrounding immune macroenvironment. The present study thus emphasizes the contribution of FTSEC function in EP and further explores more complex interaction networks to promote the development of advanced interventions for tubal rupture.

## AUTHOR CONTRIBUTIONS

Xiaoya Zhao and Qian Zhu made the single cell suspension. Xiaoya Zhao performed scRNA‐seq computational analysis and experiments. Xiaoya Zhao wrote the manuscript. Qian Zhu cultured the villous explants. Li Yan, Sifan Ji and Yiqin Zhang collected clinical samples. Lisai Ha, Chuqing He, Yuan Tian, and Luting Chen performed pathological reviews. Jian Zhang conceived this study and provided funding. Mingqing Li, Jian Zhang and Qian Zhu designed the research, revised the manuscript.

## CONFLICT OF INTEREST STATEMENT

The authors have declared that no conflict of interest exists.

## ETHICS STATEMENT

All tissue samples used for this study were obtained through the International Peace Maternity and Child Health Hospital (IPMCH) in Shanghai. The study was approved by the institutional ethics committee of the IPMCH (GKLW201909). Details about distribution of samples according to baseline characteristics are provided in the Table 1.

## PATIENT CONSENT STATEMENT

Written consents for publication were obtained from all pregnancy patients (IP, AEP, and REP).

## Supporting information


**Figure S1:** Major cell types identified by scRNA‐seq. (A) The harmony integrated data and present study visualized by UMAP. Colours indicate cluster or group. (B) Maternal–foetal interface from three groups visualized by UMAP. Colours indicate group. Violin plots showing log‐transformed, normalized expression levels to identify the 4 major clusters (KRT7, EPCAM, PTPRC and DCN). Cells from Figure 2B are used for the violin plots.
**Figure S2:** Spatially resolved developmental trajectories of EVT subsets.
(A) Differential gene pseudotemporal expression trajectory map between AEP and REP group. EVT, extravillous trophoblast. (B) Pseudotemporal ordering trajectory map (EVT1, EVT2 and EVT3). REP, ruptured tubal ectopic pregnancy. AEP, abortive tubal ectopic pregnancy. IP, intrauterine pregnancy.
**Figure S3:** Investigation of transcriptome characteristics of immune cell types and cell subtypes. (A) Dot plot presentation of scaled expression of each immune cell markers. Colours represent expression level and sizes of dot represent percentage of positive cells. (B) UMAP plots of NK subclusters. Each dot denotes one cell which is coloured by the identified subtypes. p, proliferative. (C) Violin plots of the expression of eight representative marker genes to identify the 5 major clusters. (D) Volcano plot showing DEGs (differential expressed genes) in REP compared to AEP. Blue and red represent down‐regulation and up‐regulation, respectively. (E) UMAP plots of macrophages. Each dot denotes one cell which is coloured by the groups. (F) Cell proportions of macrophages subpopulations in AEP and REP groups. Colours represent identified subtypes. M, macrophage. (G) Heat map showing relative expression of selected genes for macrophage subsets. Each column represents a single cell, and each row represents a selected gene. Colours indicate the expression levels as shown in the scale bar. (H) z‐scores of CD68, S100A9, S100A8, FCGR3A, GRP183, CCR7, CD206 and CD163 (mean expression levels) in the M1, M2 and M3. (I) Dot plot presentation of scaled expression of normal fallopian tube from Dinh et al. Colours represent expression level and sizes of dot represent percentage of positive cells. REP, ruptured tubal ectopic pregnancy. AEP, abortive tubal ectopic pregnancy. IP, intrauterine pregnancy.
**Figure S4:** Identification of fibroblasts and endothelial types at the maternal–foetal interface. (A) UMAP plots of epithelial cells. Each dot denotes one cell which is coloured by the groups. (B) Subpopulation analysis UMAP plots of fibroblasts (colours represent different subpopulations, refer to legend). ds, decidual stroma, endo, endothelia, F, fibroblasts. (C) Dot plot presentation of scaled expression of each subset markers. Colours represent expression level and sizes of dot represent percentage of positive cells. (D) GO analysis of enriched terms across feature genes of each subset. The terms with *p* < 0.05 are selectively shown. REP, ruptured tubal ectopic pregnancy. AEP, abortive tubal ectopic pregnancy. IP, intrauterine pregnancy.
**Figure S5:** CSF1+ FTSECs promote EVT invasion and induce macrophage polarize to macro2. (A) Number of significant ligand‐receptor pairs between two cell subsets. The edge widths indicate the number of ligand‐receptor pairs. (B) z‐scores of genes between the FTSECs of AEP and REP (mean expression levels). Expression values were generated using scRNA‐seq data. (C) Double immunochemical staining for PAX8 and CSF1 in the FTSECs of AEP and REP (representative images from AEP =3, REP = 3). Scale bars, 100 μm. (D) Immunochemical staining for HLA‐G (EVTs marker) and CSF1R (representative images from AEP =3, REP = 3). Scale bars, 100 μm. (E) Representative images showed the migration of HTR8/SVneo cells in the differential treatments (0 h, 18 h). CSF1 (50 ng/mL, 150 ng/mL), GW2580 (10 μM, 20 μM) and 100 ng/mL CSF1 plus 20 μM GW2580. Negative control (DMEM/F12 + 2%FBS), positive control (DMEM/F12 + 10%FBS). (F) Immunofluorescence for CD206 (macro2 marker, green) and CD80 (macro1marker, red) in macrophages. Scale bars, 50 μm. FTSECs, fallopian tube secretory epithelial cells. REP, ruptured tubal ectopic pregnancy. AEP, abortive tubal ectopic pregnancy. IP, intrauterine pregnancy.

**Table S1.** GO term analysis of the three EVT subsets.

**Table S2.** GO term analysis of the macrophage subsets.

**Table S3.** GO term analysis of the epithelial cell subsets.

## Data Availability

All single‐cell RNA sequencing data were deposited in the GEO datasets (https://www.ncbi.nlm.nih.gov/) and the GEO number is GSE207630.
