# Supplementary material for: Colony‐stimulating factor 1 positive (CSF1+) secretory epithelial cells induce excessive trophoblast invasion in tubal pregnancy rupture
Source: Cell Prolif. 2023 Jan 31;56(7):e13408. doi: 10.1111/cpr.13408 (PMC10334268; doi:10.1111/cpr.13408)
Supplement: Supplementary file 1 — Figure S1: Major cell types identified by scRNA‐seq. (A) The harmony integrated data and present study visualized by UMAP. Colours indicate cluster or group. (B) Maternal–foetal interface from three groups visualized by UMAP. Colours indicate group. Violin plots showing log‐transformed, normalized expression levels to identify the 4 major clusters (KRT7, EPCAM, PTPRC and DCN). Cells from Figure 2B are used for the violin plots. Figure S2: Spatially resolved developmental trajectories of EVT subsets. (A) Differential gene pseudotemporal expression trajectory map between AEP and REP group. EVT, extravillous trophoblast. (B) Pseudotemporal ordering trajectory map (EVT1, EVT2 and EVT3). REP, ruptured tubal ectopic pregnancy. AEP, abortive tubal ectopic pregnancy. IP, intrauterine pregnancy. Figure S3: Investigation of transcriptome characteristics of immune cell types and cell subtypes. (A) Dot plot presentation of scaled expression of each immune cell markers. Colours represent expression level and sizes of dot represent percentage of positive cells. (B) UMAP plots of NK subclusters. Each dot denotes one cell which is coloured by the identified subtypes. p, proliferative. (C) Violin plots of the expression of eight representative marker genes to identify the 5 major clusters. (D) Volcano plot showing DEGs (differential expressed genes) in REP compared to AEP. Blue and red represent down‐regulation and up‐regulation, respectively. (E) UMAP plots of macrophages. Each dot denotes one cell which is coloured by the groups. (F) Cell proportions of macrophages subpopulations in AEP and REP groups. Colours represent identified subtypes. M, macrophage. (G) Heat map showing relative expression of selected genes for macrophage subsets. Each column represents a single cell, and each row represents a selected gene. Colours indicate the expression levels as shown in the scale bar. (H) z‐scores of CD68, S100A9, S100A8, FCGR3A, GRP183, CCR7, CD206 and CD163 (mean expression levels) in [file CPR-56-e13408-s004.docx]

**
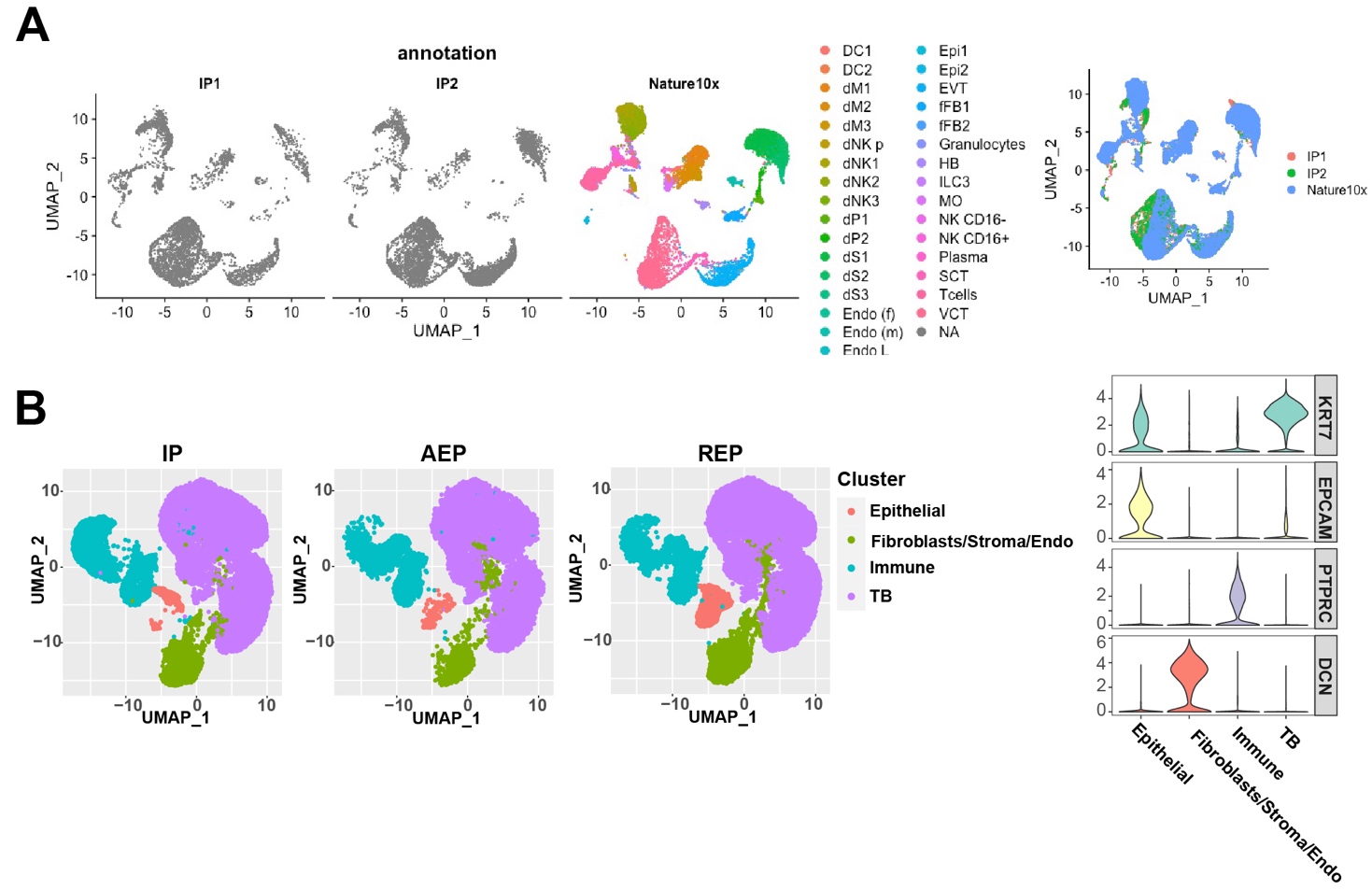
**

**Figure S1: Major cell types identified by scRNA-seq.**

A. The harmony integrated data and present study visualized by UMAP. Colors indicate cluster or group.

B. Maternal-fetal interface from three groups visualized by UMAP. Colors indicate group. Violin plots showing log-transformed, normalized expression levels to identify the 4 major clusters (KRT7, EPCAM, PTPRC and DCN). Cells from **Figure 2B** are used for the violin plots.


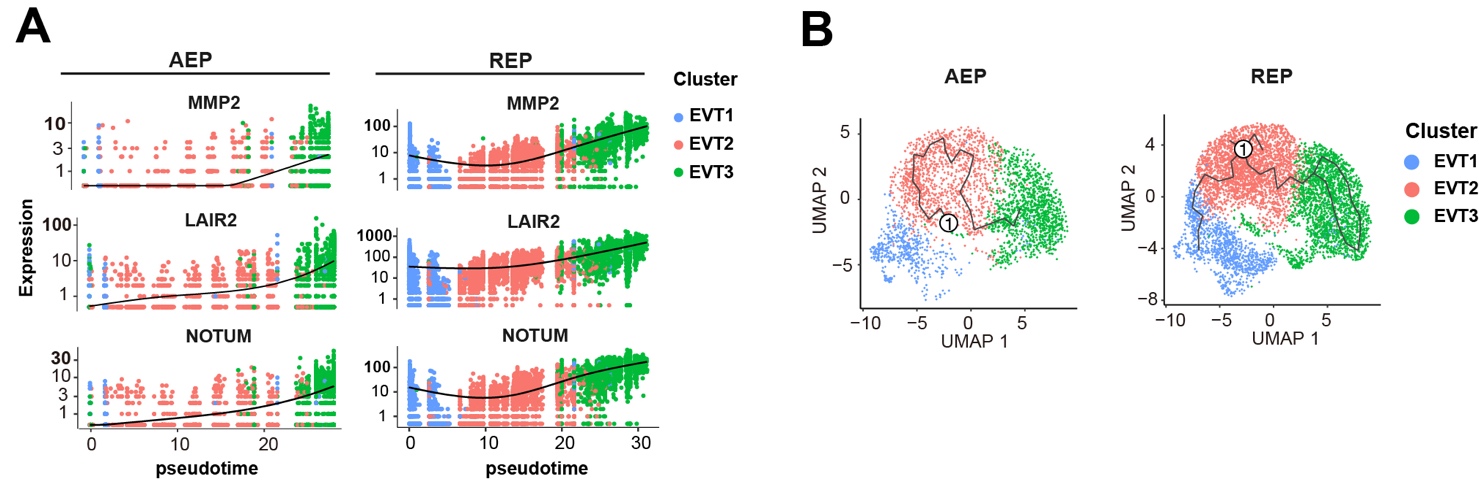


**Figure S2: Spatially resolved developmental trajectories of EVT subsets.**

A. Differential gene pseudotemporal expression trajectory map between AEP and REP group. EVT, extravillous trophoblast.

B. Pseudotemporal ordering trajectory map (EVT1, EVT2, and EVT3). REP, ruptured tubal ectopic pregnancy. AEP, abortive tubal ectopic pregnancy. IP, intrauterine pregnancy.


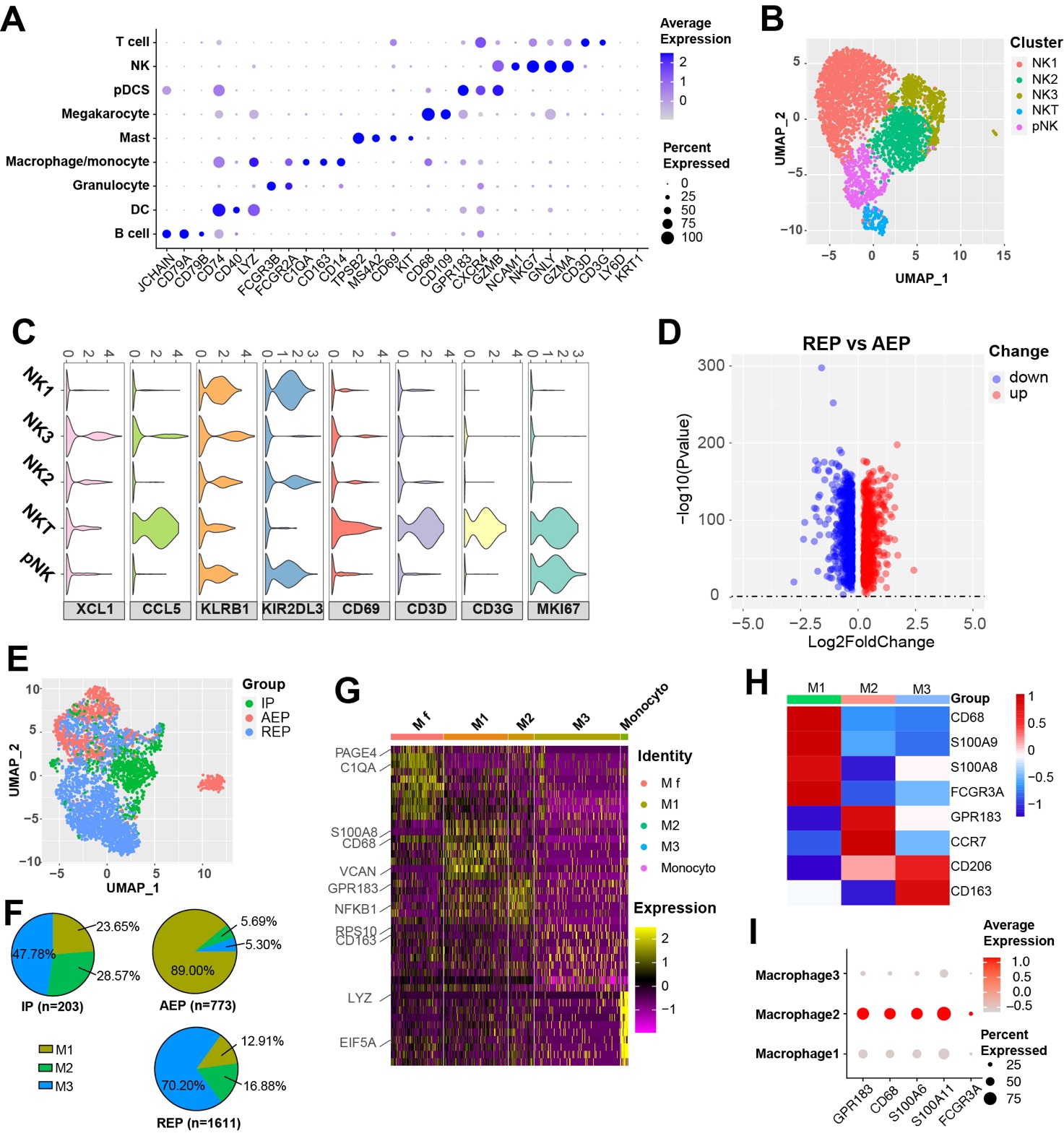


**Figure S3: Investigation of transcriptome characteristics of immune cell types and cell subtypes.**

A. Dot plot presentation of scaled expression of each immune cell markers. Colors represent expression level and sizes of dot represent percentage of positive cells.

B. UMAP plots of NK subclusters. Each dot denotes one cell which is colored by the identified subtypes. p, proliferative.

C. Violin plots of the expression of eight representative marker genes to identify the 5 major clusters.

D. Volcano plot showing DEGs (differential expressed genes) in REP compared to AEP. Blue and red represent down-regulation and up-regulation, respectively.

E. UMAP plots of macrophages. Each dot denotes one cell which is colored by the groups.

F. Cell proportions of macrophages subpopulations in AEP and REP groups. Colors represent identified subtypes. M, macrophage.

G. Heat map showing relative expression of selected genes for macrophage subsets. Each column represents a single cell, and each row represents a selected gene. Colors indicate the expression levels as shown in the scale bar.

H. z-scores of CD68, S100A9, S100A8, FCGR3A, GRP183, CCR7, CD206, and CD163 (mean expression levels) in the M1, M2, and M3.

I. Dot plot presentation of scaled expression of normal fallopian tube from Dinh et al. Colors represent expression level and sizes of dot represent percentage of positive cells. REP, ruptured tubal ectopic pregnancy. AEP, abortive tubal ectopic pregnancy. IP, intrauterine pregnancy.


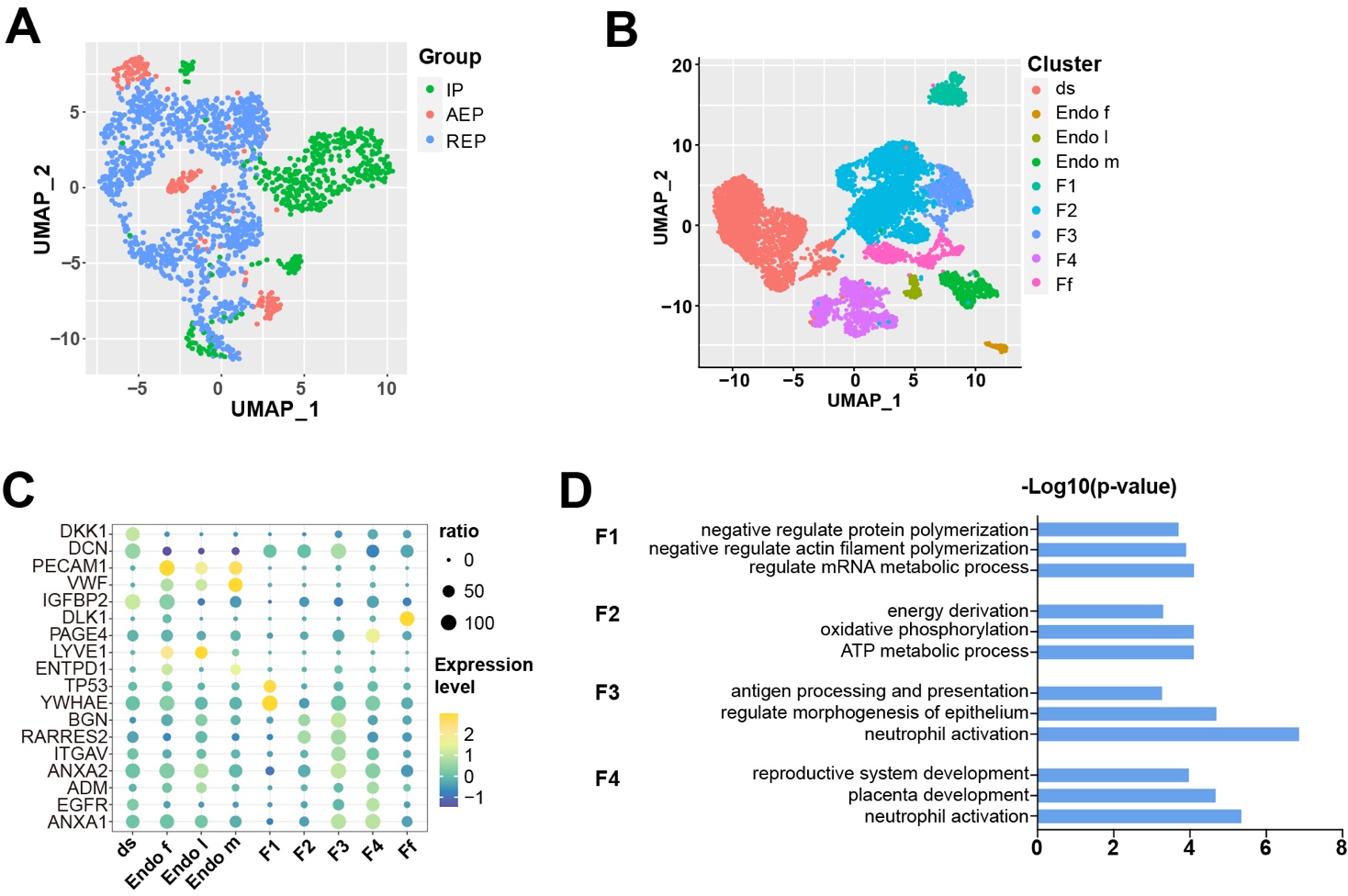


**Figure S4: Identification of fibroblasts and endothelial types at the maternal–fetal interface.**

A. UMAP plots of epithelial cells. Each dot denotes one cell which is colored by the groups.

B. Subpopulation analysis UMAP plots of fibroblasts (colors represent different subpopulations, refer to legend). ds, decidual stroma, endo, endothelia, F, fibroblasts.

C. Dot plot presentation of scaled expression of each subset markers. Colors represent expression level and sizes of dot represent percentage of positive cells.

D. GO analysis of enriched terms across feature genes of each subset. The terms with p < 0.05 are selectively shown. REP, ruptured tubal ectopic pregnancy. AEP, abortive tubal ectopic pregnancy. IP, intrauterine pregnancy.


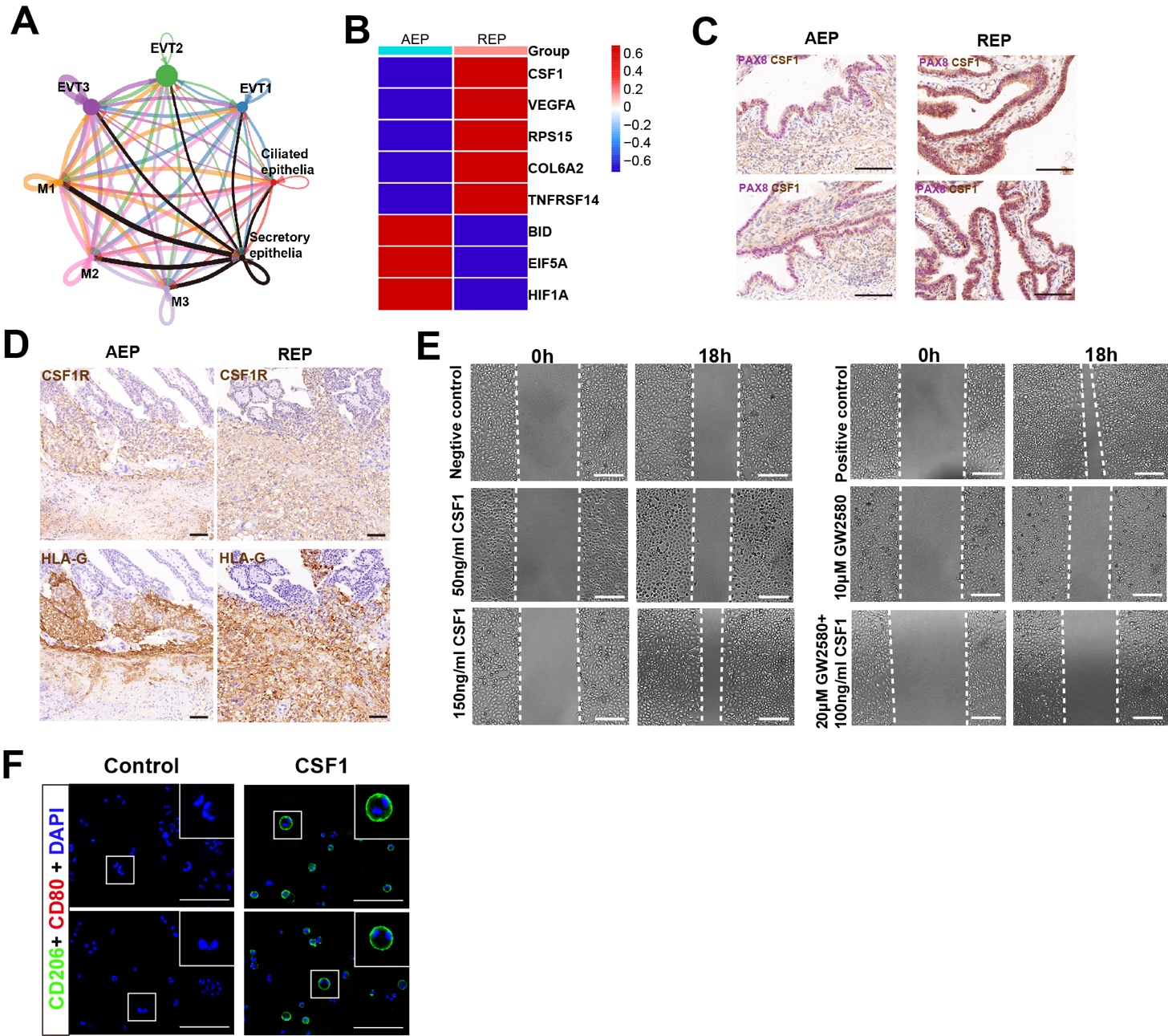


**Figure S5: CSF1+ FTSECs promote EVT invasion and induce macrophage polarize to macro2.**

A. Number of significant ligand-receptor pairs between two cell subsets. The edge widths indicate the number of ligand-receptor pairs.

B. z-scores of genes between the FTSECs of AEP and REP (mean expression levels). Expression values were generated using scRNA-seq data.

C. Double immunochemical staining for PAX8 and CSF1 in the FTSECs of AEP and REP (representative images from AEP =3, REP = 3). Scale bars, 100μm.

D. Immunochemical staining for HLA-G (EVTs marker) and CSF1R (representative images from AEP =3, REP = 3). Scale bars, 100μm.

E. Representative images showed the migration of HTR8/SVneo cells in the differential treatments (0h, 18h). CSF1 (50ng/ml, 150ng/ml), GW2580 (10µM, 20µM), and 100ng/ml CSF1 plus 20µM GW2580. Negative control (DMEM/F12 + 2%FBS), positive control (DMEM/F12 + 10%FBS).

F. Immunofluorescence for CD206 (macro2 marker, green) and CD80 (macro1marker, red) in macrophages. Scale bars, 50μm. FTSECs, fallopian tube secretory epithelial cells. REP, ruptured tubal ectopic pregnancy. AEP, abortive tubal ectopic pregnancy. IP, intrauterine pregnancy.
